# Supplementary material for: Adverse childhood experiences and elder abuse victimization nexus: A systematic review and meta-analysis
Source: PLoS One. 2025 Feb 5;20(2):e0316902. doi: 10.1371/journal.pone.0316902 (PMC11798435; doi:10.1371/journal.pone.0316902)
Supplement: Attachment — Submitted filename: Response to Reviewer.docx [file pone.0316902.s003.docx]

Dear Kristen

Thank you very much for the opportunity to respond to the reviewer’s comments. We want to emphasize that the suggestions made by the reviewers have improved the manuscript. We have addressed the reviewer’s comments below:

**Response to Reviewer’s comments**

Please address Reviewer 1 numbered comments as follows:

1. **Provide a more concise overview of the findings in the abstract.**

**Response:**

A more concise abstract has been provided.

2. **On Page 4: it is stated that “… the prevalence of abuse in many countries, especially low- and middle-income countries is expected to increase with the increasing rate of population ageing (WHO, 2022)”. It is unclear why the issue is especially relevant for low- and middle-income countries? There is no clear explanation even after checking the citation.**

Response:

Thank you for this comment. I have modified the sentence and included another reference and it now reads as “**In addition to the reported increase of abuse during the Coronavirus disease – 2019 (COVID-19) (Chang, 2016), the prevalence of abuse in many countries, including low- and middle-income countries is expected to increase with the increasing rate of population ageing (World Health Organization, 2024; Yon et al., 2017).”** The article published by WHO in 2022 was based on one of the studies which is a systematic review and meta-analysis, reporting a high prevalence of elder abuse in low- and middle-income countries as well. Out of the 28 studies included in the systematic review, 7 were based in low- and middle-income countries.

3. **Provide a citation supporting the claim of a higher prevalence of EAV in low- and middle-income countries compared to higher income countries.**

**Response:**

Thank you. The two citations (World Health Organisation (2024) and Yon et al., (2017) have been provided.

4. ***The sentence in question reads as if there is only one pathway from ACEs to EAV, which is not true. Please revise this sentence to reflect that the pathway described is one of several potential pathways, of particular relevance to the present study, and provide relevant citations.***

**Response:**

The sentence has been revised and referenced. It now reads as **“**One of these pathways explored in this study is that older adults who experience adverse circumstances in their childhood, living in any setting, may carry disempowerment with them throughout later life, and are more likely to be re-victimized in the community or when living in a residential setting (Asyraf et al., 2021)”

5. ***I believe this paragraph is relevant. However, please shorten and focus on other systematic reviews linking ACEs to other outcomes and to the highlighted claim regarding the absence of systematic reviews on ACEs and EAV.***

Response:

Thanks for this suggestion. I have made the argument more concise. It now reads as “For instance, systematic reviews have provided evidence on ACEs, or effects of multiple ACEs on health outcomes such as sexual, mental, and physical health, and higher utilisation of healthcare (Hughes et al., 2017; Kalmakis & Chandler, 2015; Petruccelli et al., 2019)

6. ***Please be sure to temper the claim that there has been "a significant number of bodies of research on this association". The outcome of the systematic review itself suggests that this is not the case and contradicts a later claim that there is a "low volume" of scholarship on this topic in research (Critical finding, page 20).***

Response:

We have acknowledged this discordance and addressed it accordingly. The claim now reads as “Moreover, following an emerging recognition of the impact of ACEs on EAV in later life, there have been some papers published on the association (Burnes et al., 2022; Easton & Kong, 2021; McDonald & Thomas, 2013; Wiklund et al., 2022)

7. ***Please thoroughly review the submission for typos.***

Response

We have read the entire manuscript for typo corrections.

*7****. The criteria are met for a systematic review.***

**Response**:

Thank you very much for this comment. We appreciate this feedback.

***8. In discussion, I recommend expanding the discussion about measurement consistencies: how they are inconsistent and what are the problems associated with the inconsistent measurement. Also, the discussion about moderating factors should be approached carefully since they were based on two studies only***

Response:

We have expanded the discussion on measurement and their potential impacts. Also, we have modified our discussion on the moderating factors elaborating on the possible implications for research, policy and practice.

***9. Please expand the discussion on the justification for applying a life course perspective, as described by the Reviewer. On page 19: I recommend expanding the discussion about ‘the association between ACEs and EAV aligns with the life course perspective’. How does it align and are there other theories that can explain the association? Authors can consider bringing in the prior literature about lifetime revictimization. The discussion about the strengths and limitations of the study was very brief, which needs to be strengthened and clearly specify the contribution of this study.***

**Response:**

Thank you for the suggestions to elaborate on the justification for applying the life course perspective. We have provided relevant information including the justification for the life course perspective in the introduction making it easier for the reader to understand the discussion of the life course perspective in the discussion section.

***10. Be sure to address the comments of Reviewer 1 in terms of what to emphasize in more detail. I would also like to see a brief mention of possible limitations of excluding grey literature from the search process.***

**Response:**

Thank you for these comments. I have included in the discussion that, “Moreover, though there was no study excluded based on grey literature status, the inclusion of this as an exclusion criterion might have automatically excluded grey literature that reported on the association between ACEs and EAV in the first place.”

Please address Reviewer 2 comments as follows:

***I agree that the inclusion of ageism as a form of EAV could lead to inflated rates of EAV, at least in terms of how it is typically conceptualized. Please elaborate on the motivation for its inclusion and if possible, provide the number of articles included in the review that were based only on the link between ACEs and ageism in populations 60 and over. If one or more were included based on only this specific association, please discuss the implications of including it on the overall findings from the review.***

Response:

Thank your for your comment on EAV and ageism. **Ageism** is a bias against older adults groups based on their age, which may take the form of discrimination at all levels against such individuals and groups, up to and including victimisation and bullying (Pillemer, Burnes, & MacNeil, 2021). Investigating the connection between ageism and elder mistreatment. *Nature Aging*, *1*(2), 159-164.). In view of this, we included ageism as a synonym for elder abuse victimisation. We want to profoundly say that none of the studies included specifically measured the association between ACEs and ageism.

***2. Inclusion and exclusion criteria should offer more details, as noted by the Reviewer. As it relates to study eligibility for inclusion in the review, did you consider qualitative versus quantitative studies, year of publication, location of study, or type of analysis that examined the relationship between ACEs and EAV?***

Response

We have provided more information on the inclusion strategies. Under the “study eligibility criteria”, we have included that the Inclusion of studies was not restricted to any research approach, year of publication, location of study, or type of analytical method used to examine the association on ACEs and EAV. These inclusion strategies allowed for any study available on the ACEs – EAV association to be included in the systematic review.

***3. Please address the Reviewer's question about whether inclusion required a finding of a statistically significant relationship.  To be included in the review, did the study need to have reported a significant association between ACEs and EAV, or did it also include studies that found no significant relationship?***

Response:

The significance level as reported by the study did not determine its inclusion in the systematic review. The most relevant inclusion indicator was for the study to report examining as association between ACEs and EAV.

***4. Please review the details offered about specific studies to ensure accurate reporting. The authors are encouraged to review the accuracy of information captured on studies included in the review. For example, the Burnes et al (2022) study did not use a three items questionnaire to measure EAV, nor were there 36 items included in the child maltreatment questionnaire.***

Response:

We have reviewed the included studies for accurate reporting.

***5. Please clarify what is meant by cumulative EAV.***

**Response:**

We have replaced the cumulative EAV with ‘multiple’ to represent an experience of more than one elder abuse victimisation. This could include experiencing say **emotional abuse** and **physical abuse** together, or **emotional abuse, physical abuse** and **financial abuse.**

*6. Please review the conclusion section after making the revisions outlined above and add other relevant takeaways, if warranted.*

Response:

Thank you very much. We have reviewed the conclusion of the review to capture all important take home messages.
