# Supplementary material for: Adverse childhood experiences and elder abuse victimization nexus: A systematic review and meta-analysis
Source: PLoS One. 2025 Feb 5;20(2):e0316902. doi: 10.1371/journal.pone.0316902 (PMC11798435; doi:10.1371/journal.pone.0316902)
Supplement: Attachment — Submitted filename: Responses to Revision 1 ACES and EAV.docx [file pone.0316902.s005.docx]

**Responses to Comments**

Dear Editor,

We thank you very much for the comments raised, which have improved the paper immensely. We have addressed the comments in the manuscript and also below;

**Journal Requirements:

Please review your reference list to ensure that it is complete and correct. If you have cited papers that have been retracted, please include the rationale for doing so in the manuscript text, or remove these references and replace them with relevant current references. Any changes to the reference list should be mentioned in the rebuttal letter that accompanies your revised manuscript. If you need to cite a retracted article, indicate the article’s retracted status in the References list and also include a citation and full reference for the retraction notice.

Response**

**I have reviewed the reference list for accuracy. The reference is complete and correct.**

**Additional Editor Comments:

This is a strong paper that makes an important contribution to the research literature. While you sufficiently addressed reviewers’ concerns in this revision, a thorough editing of the manuscript for grammar and syntax was not apparent. PLOS ONE does not provide on-staff editorial assistance, so the job of an editorial board member includes reviewing manuscripts for grammar, punctuation, syntax, and clarity consistent with the journal’s requirements. In future submissions, please take this into account as it will significantly speed up the review process. While I have many comments, they are all minor and require a wording change or attention to the use of singular vs. plural nouns, use of commas, and the like. I have uploaded my Word document with my editorial changes and additional comments to make your review of my changes easier to work**

**Response**

**Thank you very much for the thorough proofreading of the manuscript. We have addressed all comments raised in the manuscript.**

**General comments:**

1. Use “in singular or multiple form” rather than “by form or multiple” throughout manuscript. Example from the abstract: “All studies that addressed associations between ACEs, ~~by form or multiple~~ **in singular or multiple form**, and EAV were included in the review.” I note that you did attend to this prior comment from me in this revision, but in some instances in the manuscript, the word “cumulative” still appears. I note that measures capturing multiple forms of ACEs or EAVs can be measured dichotomously (e.g., having multiple vs. none or having particular combinations), and not necessarily cumulatively (i.e. a sum or count), so use these two terms with intentionality. For example, when I see the word “cumulative”, I expect the model coefficient for a predictor to represent the predicted change in an association for each additional number of something (e.g., ACEs) included in a count variable. It may be that you have intentionally chosen to use one of these terms over the other—I am just requesting that you double-check and where appropriate, use consistent terminology.

**Response**

I have perused the entire manuscript and now consistent with the use of “in singular or multiple form”.

2. Once you spell out “adverse childhood experiences” within the main text following the abstract, and give the acronym “(ACEs)”, you can use ACE throughout the manuscript. If you choose to use the fully spelled out term interchangeably throughout, then, at a minimum, be consistent in subsection titles (use “ACEs” or use “adverse childhood experiences”).

**Response**

This has been taken care of in the entire manuscript.

3. Check that singular vs. plural is used correctly (e.g., in the abstract, “odds ratios (OR)” is plural but “confidence interval (CI) is singular. When referring to one as plural, the other should be plural.

**Response**

I have corrected this in the abstract and in the manuscript as well.

4. Check appropriate use of commas throughout manuscript. Some, but not all, examples are given below.

When you refer to EAV, you sometimes use EAVs. It would sound strange to say “elder abuse victimizations” if you are simply referring to any form of EAV. Please check throughout the document to make sure you intend to use a plural form of EAV.

**Response**

I have corrected this problem in the entire manuscript.

5. Check for unnecessary double-spacing *within* sentences.

**Response**

I have removed all unnecessary spaces within the manuscript.

6. **The comments above should be addressed throughout the manuscript. In some comments below, I have identified where these types of changes should be made, but not all instances were flagged.**

**Abstract:**

**Add an “s” to “association”: Included studies examined multiple ACEs and multiple EAV associations (N=3)**

**Awkwardly worded sentence: The results indicate that interventions designed to reduce ACEs, ~~by form or multiple~~ in singular or multiple form, early in life and to support residential-and community-dwelling older adults may be relevant in reducing the incidence of EAV**

**“The life course perspective should be integrated….”**

**Response**

I have addressed all of these comments raised here in the manuscript.

**Background**:

**(Page 3)**

7. **Make “pose” plural: “The growing prevalence and incidence of elder abuse victimization (EAV) reported in residential and community (home care) settings across the world poses both a public health and a human rights concern.”**

**Add comma after second use of the word “countries”: “…the prevalence of abuse in many countries, including low- and middle-income countries, is expected to increase…”**

**Change wording: “Increasing levels of elder abuse co-occur with high levels of child maltreatment and adverse childhood experiences (ACEs) (Madigan et al., 2023).”**

**Response**

We have rectified the above issue in the manuscript.

8. **Since the word “adults’ “ is a possessive plural, delete “the” and make “experience” plural: “EAV, in this review, will refer to ~~the~~ older adults’ experiences of a single or repeated act or lack of appropriate action…”**

**Add comma after “knowledge”, change “enunciate” to “elucidate”: “However, to our knowledge, no systematic syntheses or reviews currently exist to ~~enunciate~~ explore the nature of this connection/pathway”.**

**(Page 4)**

**“Specific examples of ACEs include childhood sexual abuse, parental criminality and divorce, childhood disempowerment, and childhood discrimination (Hughes et al., 2017).”**

**What does “This” refer to?: “This includes the cumulative harmful effects of at least two domains of ACEs (Hughes et al., 2017).”**

**Response:**

We have addressed all the above comments in the manuscript.

9. **In the following sentence, change “these” to “the” since you are referring to the singular noun “One”: “One of the~~se~~ pathways explored in this study…”**

**Change wording: “~~There has been research~~ Studies on the associations between ACEs and adverse outcomes~~, with use varying~~ vary in their designs, methodologies, locations, findings and conclusions.”**

**Drop the “s” in “influences”: “For example, a study conducted in China revealed that social and economic instability during childhood influence~~s~~ an increase in…”**

**Add comma after second “ACEs”: “For instance, systematic reviews have provided evidence on ACEs, or effects of multiple ACEs, on health outcomes such as…”**

**Simplify sentence: “~~Moreover, following an emerging recognition of the impact of ACEs on EAV in later life, there have been some~~ In addition to an earlier study (McDonald & Thomas, 2013), more recent research has focused on the association between ACEs and EAV (Burnes et al., 2022; Easton & Kong, 2021; ~~McDonald & Thomas, 2013;~~ Wiklund et al., 2022).**

**Response**

The above comments have been addressed in the manuscript.

**(Page 5):**

10. **Change wording: “Synthesizing evidence on this association will ~~be significant in~~ contribute to understanding, or revealing, a clear pathway of the association and evidence on how to mitigate this public health concern.”**

**Change wording: “~~In this study,~~ As applied elsewhere (McDonald & Thomas, 2013), we employed the life-course perspective as a useful framework ~~to understand~~ for interpreting the findings of the systematic review ~~as applied elsewhere (McDonald & Thomas, 2013)~~…”**

**Use plural for “experience”: “…about aspects of developmental experiences…”**

**Add “a” or “the”: “According to Elder (2006), a life-course perspective elucidates the potential effects of accumulative advantage or disadvantage of individuals – social structure relationship on individuals over time.”**

**Drop this sentence or elaborate on this example and provide a citation: “To illustrate this, the experience of a child living in an institution such as a school or a family setting will continue to be important in later life.”**

**Combine sentences: “~~Accordingly,~~ This systematic review aimed to understand the connections between ACEs and EAVs, as well as ~~. We also need to~~ gain insight into factors moderating the relationships between ACEs – EAVs ~~relationships~~.**

**Change wording: “~~Understanding~~ An enhanced understanding of this evidence using a life-course perspective would assist in the development of appropriate interventions for older adults who experience abuse, prevent the occurrence of ACEs~~through the understanding of life-course perspectives~~, and ~~provide an urgent and clear a clearer pathway for more~~ improve cross-cultural and longitudinal research on the relationships between ACEs and EAV.”**

**Response**

All the comments raised above have been addressed in the manuscript.

**(Page 6):**

11. Change wording: “Two authors (KAN and KAC) did the screening independently and met on Zoom to address any disagreement ~~for~~ **and achieve** consensus.”

Change wording: “Studies on elder abuse victimization, or aspect on elder abuse types, such as ageism, psychological abuse,…”

Change wording: “~~Inclusion of studies were~~ **Study inclusion was** not restricted…”

Change wording; add comma after “manager”, remove comma after “removal”: “Articles ~~that resulted from the~~ **identified in** databases search**ed** were exported to Endnote, a reference manager**,** to allow for duplicate removal~~,~~ and title and abstracts screening, as per the study’s inclusion criteria.”

**Response**

**All comments above have been addressed in the manuscript.**

**(Page 6-7):**

Remove comma after “retrieved”; change wording: All articles that potentially met eligibility criteria were retrieved~~,~~ after ~~the~~ **a** full test screen of all potential articles ~~was conducted~~. ~~Afterwards~~ **After consulting with the other three authors,** all articles meeting the eligibility criteria were retained. ~~After consulting with the other three authors.~~

**Response**

**We have addressed the above comments.**

**(Page 7):**

12. Add comma after “August 2023”: “The search was conducted in English and performed in PubMed, CINAHL Complete, APA PsycINFO, Web of Science, Scopus, and Google Scholar in August 2023**,** covering articles published in or before 2023.”

**Respone**

**We have addressed the above comment in the manuscript.**

**(Page 8):**

13. **Add comma before “respectively”: The checklists were made up of 8 and 11 questions for analytical cross-sectional and cohort studies, respectively.**

**Change wording: “After eligible studies ~~had been included~~ were identified,…”**

**Should “structures” be singular?: “This grouping generated themes providing structures for the presentation of the findings.”**

**Response**

We have addressed comments above in the manuscript.

**(Page 9):**

14. **Make “interval” plural”: After the narrative review was completed for a particular theme, a meta-analysis of the extracted odds ratios and confidence intervals.”**

**Add comma after ratios and drop the word “and”: “Pooled odds ratios, ~~and~~ confidence intervals and heterogeneity were used to present consolidated associations for the themes identified, where applicable.”**

**Change wording: “After title and abstract screening ~~had been~~ was completed…”**

**Change wording: “The full text screening resulted in a total of 9 articles (all employed quantitative methods) that met eligibility criteria for the systematic review…”**

**Change wording: “None ~~was~~ were conducted in an African country.”**

**Response**

The above comments have been addressed in the manuscript.

**(Page 10):**

15. **Change wording and address plural/singular and tense corrections: “The sample sizes of the included studies ranged~~s~~ from 135 to 23,468, ~~with the~~ and participants were ~~being~~ predominantly females ~~in all included studies~~.”**

**~~“Participants in the included studies were aged 60 years and above, who reported ACEs and EAV experiences in later life.”~~**

**Change wording: “Some ~~of the~~ studies measured ACEs cumulatively ~~on cumulative~~ (Asyraf et al., 2021; Chen & Fu, 2022b; Dong & Wang, 2019; Easton & Kong, 2021) while~~s~~ others ~~used a~~ measured types of ACE separately (Burnes et al., 2022; Giraldo-Rodríguez et al., 2022; Kong & Easton, 2019; McDonald & Thomas, 2013; Wiklund et al., 2022) ~~to measure childhood experience~~.”**

**Change wording: “The studies reported using different types of measurement tools with unequal and unique sets of questions to measure ACEs.”**

**Change wording: “….and a questionnaire measuring ~~an~~ abuse before the age of 65. ~~Years~~ ~~questionnaire~~**

**Delete sentence as it is redundant with first sentence in the paragraph: ~~“All of these questionnaires were used to measure ACEs either on cumulative or in part.”~~**

**Response**

We have addressed all the comments above in the manuscript.

**(Page 11):**

Change wording: “Even though cumulative EAV ~~(cumulative)~~ was the ~~main~~ **the most common** outcome measure **among the studies** (Asyraf et al., 2021; Burnes et al., 2022; Chen & Fu, 2022b; Easton & Kong, 2021; Kong & Easton, 2019), ~~other~~ **single forms of EAV** ~~outcomes~~ ~~contributing to our understanding of EAV~~ were reported**, as well, including** ~~These single outcome measures included experience of”~~ physical elder abuse…”

Change wording: “The included studies used distinct scales **and question items** to measure EAV, including ~~The included studies used sets of questions including~~ the Abusive Behavior Inventory…”

Check official name of measure, as I’ve only seen it referenced with “scales” capitalized and singular: “…Revised Conflict Tactics scales (Asyraf et al., 2021)…”

Change wording in section title: Is it “multiple” or cumulative”?—be sure you are using these terms purposively. “Multiple” forms could be operationalized in a non-cumulative way (e.g., a dichotomous variable). If you always mean cumulative, then section title should use that word instead of “multiple”; either capitalize each separate word in ACEs or use small case for “adverse” in section title; also, “experience” should be plural, correct?: “Connections between Adverse childhood experience**s** and multiple elder abuse victimization”

Change wording and check whether “multiple” should be “cumulative” and the reverse: ” Seven studies examined ~~the~~ association between ACEs (multiple or single forms) ~~or part~~ and cumulative EAV.”

**Response**

**The comments have been addressed in the manuscript.**

**(Page 12):**

Delete “in”: “Burnes et al. (2022) and Dong and Wang (2019) reported that a history, or increasing incidence of ~~in~~ child maltreatment…”

Include direction (e.g., “positive” association) of the relationship: “Similarly, ACEs had a significant association with EAV (Easton & Kong, 2021).”

Change wording; also, should “ACE” be plural here? If not, change to “at least one ACE and”: “The pooled odds ratio from a meta-analysis shows a statistically significant association between experiencing ACE**s** and multiple EAV…”

Change wording: “Some ~~of the~~ studies examined the effect of having a history of a co-occurring abuse/ACEs on EAV in later life. While most of the results were consistent, some contradictions were apparent. Experiencing either three or at least four ACEs was associated with EAV (Asyraf et al., 2021; Chen & Fu, 2022b; Giraldo-Rodríguez et al., 2022). While a significant association with EAV was also suggested when a child experienced one or two ACEs (Giraldo-Rodríguez et al., 2022), Asyraf et al. (2021) ~~experienced~~ **found** no significant association.

Clarify the implication of this statement: “Despite this, heterogeneity existed across studies…”

**Response**

**The above comments have been addressed in the manuscript.**

**(Page 13):**

Use “While” not “Whiles”: “While~~s~~ in the Mexican study….”

Consider simply saying the following: “In both ~~a similar geographic contexts (~~Mexico and **the** US~~)~~, childhood sexual abuse was significantly associated with EAV in later life (Giraldo-Rodríguez et al., 2022; Kong & Easton, 2019).

Some items not described in search strategy, starvation and frequent quarrels, seem to appear without justification. Particularly because they do not have statistically significant associations with EAV, consider dropping these items from the sentence. If they were, in fact, part of an intentional search strategy, please clarify that earlier in the manuscript when describing search terms: “~~Starvation in childhood,~~ **P**arental divorce during childhood ~~and frequent quarrels between parents individually~~ did not have ~~any~~ a significant association with EAV in later life (Chen & Fu, 2022b).”

Use plural for “experience” in section title: “Connections between adverse childhood experience**s** and ~~any one type~~ single forms of elder abuse victimization”

Combine sentences: “Overall, two of the studies examined the association between ACEs and any one type of EAV ~~(Asyraf et al., 2021; McDonald & Thomas, 2013). Both~~ and both ~~studies~~ reported that ~~was~~ ACEs were associated with the risk of experiencing any one type of EAV in later life (Asyraf et al., 2021; McDonald & Thomas, 2013).

**Response**

**The above references have been addressed in the manuscript.**

**(Page 14):**

Use plural for “experience”: “Specifically, a study conducted in Canada reported that an increase in ACE levels had significant positive association with financial elder abuse experience**s** in later life (Burnes et al., 2022).”

“Similarly, but comprehensively, a Malaysian study further found that experiencing two or three ACEs was associated with financial elder abuse in later life (Asyraf et al., 2021).”

Use plural form of “experience” or delete the word: “In China, a study that measured the association between child maltreatment – a measure of ACE – reported that an increase in child maltreatment levels was associated with an increased risk of financial elder abuse ~~experience~~ in later life (Chen & Fu, 2022a).”

Use plural form or “ratio”: “When the odds ratio**s** were pooled in a meta-analysis…”

Delete “experience”: “…(Fig 6), there was a statistically significant association between ACEs and financial elder abuse ~~experience~~ in later life...”

Clarify wording—either delete “a” or add something after “heterogeneity” to clarify that it is an actual test or statistic: “…with ~~a~~ significant heterogeneity across studies…”. Check this use of wording elsewhere in the document, too.

Use plural for ACE in subsection title and throughout this paragraph: “Connections between ACE**s** and physical abuse experience in later life”. A reminder to also check throughout the document that you are using ACEs when referring to all or multiple forms of ACEs.

**Response**

**The above references have been addressed in the manuscript.**

**(Page 15):**

Delete word or provide further rationale for it: “The Asyraf et al. (2021) study in Malaysia ~~vividly~~ reported…”

Delete words: “~~as a child with~~ a childhood experience of four or more ACEs was associated with physical elder abuse (Chen & Fu, 2022b).

Delete “experience” and check the language related to “heterogeneity”, as discussed above: “The pooled odds ratio in a metaanalysis (Fig 7) showed a statistically significant association between ACEs and physical elder abuse ~~experience~~ in later life (OR=1.01, 95%CI: 1.01, 1.02), with a significant heterogeneity across studies (I 2=81.9%; p<0.05).

Make “ACE” anbd “experience” plural in subsection title: “Connections between ACE**s** and emotional, psychological, and sexual elder abuse experience**s** in later life”

Delete unnecessary comma and change wording: “One of these studies~~,~~ reported that an increase**d** incidence of childhood maltreatment ~~increased~~ elevated the risk of emotional elder abuse experienced in later life (Chen & Fu, 2022b).

Delete extra period: For Asyraf et al. (2021) reporting three or more ACEs was associated with psychological elder abuse in later life. ~~.~~

**Response**

**The above comments have been addressed in the manuscript.**

**(Page 16):**

Delete numeric “9”: “We sourced the data from ~~9 (~~nine~~)~~ …”.

Remove yellow highlight for the word “The” at the beginning of the second paragraph; combine sentences: “The results further show different measures of ACEs and EAV, raising questions about **the reliability and validity of measures measurement** ~~consistencies~~ (Burnes et al., 2021; Dong, 2015). ~~The differences and inconsistencies in the measurements of ACE and EAV cast doubt on the reliability and validity of measures~~

Change wording: “For instance, only one study ~~that~~ used a standardized instrument (International questionnaire (ACE-IQ)) to measure ACE – ACEs (Asyraf et al., 2021), ~~with~~ **whereas** the ~~rest of the~~ **remaining** studies used different instruments to measure an aspect of ACEs such as childhood neglect, physical and sexual abuse (Giraldo-Rodríguez et al., 2022; Kong & Easton, 2019).”

Check capitalization of “scales”, as noted above: “…Revised Conflict Tactics scales…”

Add comma after “inconsistencies”; change wording: “Due to these inconsistencies, comparisons of study findings across time and space ~~might be~~ **are more** difficult to achieve, warranting the need for a standardized tool that can be used by all researchers to understand ~~the~~ **associations between** ACEs **and adverse experiences later in life**~~experience across different settings~~.

**Response**

**The above comments have been addressed.**

**(Page 17)**:

Change wording: “Our results further show that gender, physical health, and depressive symptoms ~~are~~ ~~some of the~~ moderat**e**~~ors~~ ~~of~~ the association between ACE and EAV.

Change wording: “Specifically, an older woman with an experience of multiple ACEs or any **one** form **of ACE** are more likely to experience EAV in later life.

Change wording: “Likewise, living with ~~a~~ poor physical health with an experience of multiple ACEs or any **one** form **of ACE** ~~is a more likely to~~ **increases the likelihood of** experienc**ing** EAV in later life.

Change wording: “Moreover, expressing depressive symptoms and ~~had~~ ~~a~~ **having** previous ~~experience of~~ multiple **or single** ACEs ~~or any form~~ ~~possess~~ **increase** the risk of experiencing EAV”

Change wording: “Even though the present study has shown some evidence ~~of the~~ that **gender, physical health status, and presence of depressive symptoms** moderate the ACEs – EAV association, ~~being moderated by gender, physical health status, and presence of depressive symptoms,~~ **only two studies were identified that support these findings**. **T**hese **moderated associations** should**, therefore,** be considered by researchers, policymakers and practitioners with care ~~as only two studies reported this evidence~~.

Change wording: “The~~is~~ paucity of evidence to ~~cement~~ **support** the moderating powers of gender, physical health status, and depression on the ACEs – EAV association calls for ~~enormous~~ **significantly greater attention** to research from different geographical settings. Irrespective of the fact that this evidence has not been **firmly** established, interventions tackling EAV should ~~also take interest in~~ **attend to the unique needs of** women/girls, those living with poor physical health, and **those** with depression.

Change wording: “Theoretically, the association between ACEs and EAV aligns with the life-course perspective, which argues that events ~~of~~ **occurring at** different stages of life…”

(Page 18):

Change wording: “From a life course perspective, the association between ACEs and EAV reinforces the need for ~~preventive actions~~ **interventions** in childhood ~~through child protection interventions~~ **to prevent the occurrence of ACEs and mitigate adversities stemming from ACEs throughout life.**

Change wording: **“**The systematic review and meta-analysis have ~~some~~ strength**s** and limitations ~~that need to be acknowledged~~. ~~First of all the~~ **A key** strength **is the consolidation of** ~~this academic piece has consolidated the~~ research on the strength and nature of association**s** between ACEs and EAV. ~~making it possible for policy makers, practitioners and researchers to implement the findings of the study.~~ The current study ~~will~~ can ~~serve as a base study for~~ **motivate and** **inform the design of** more longitudinal studies to examine ~~the trends of the~~ associations between ACEs and EAV, in part or multiple, **as well as ~~their~~ moderating factors in these relationships~~,~~** ~~to understand the specific intervention needed to address the changing needs of older adults and children~~. ~~Some limitations ought to be reported and discussed~~ **A key limitation is the small number of studies eligible for inclusion in this study.** Though ~~9~~ nine studies ~~making up a~~ **collectively comprise** a total sample of 60,365 ~~are good sample~~, a larger number of studies **could** have **likely** produced more **robust findings**~~produced more significant and established evidence~~. Moreover, though there was no study excluded based on grey literature status, ~~the inclusion of~~ ~~this as an~~ exclusion criterion might have automatically excluded grey literature ~~that reported on the association between ACEs and EAV in the first place~~ **relevant to this analysis**. ~~creating a gap for researchers to review grey literatures to understand their contributions on the ACEs – EAV associations~~

**Response**

**The above comments have been addressed in the manuscript.**

**(Page 19):**

Change wording and move one sentence: “There is growing evidence of an association between ACEs and EAV moderated by ~~potential confounders such as~~ gender, and physical and mental health. However, the scope of the conversation is limited to the number of available published research evidence and the global North, calling for more longitudinal studies on ACEs – EAV associations and moderating factors in different geographical contexts including developing countries.

Change wording: “~~Evaluating the 9 included papers, we considered them as~~ **A low volume of research on the association between ACEs and EAV** limit**s**~~ed~~ scholarly **discussion on this topic** ~~conversation on the association between ACEs and EAV~~ • ~~There is no~~ **C**onsistency in the measurement of ACEs and EAV **is lacking**, raising questions of measurement consistencies and comparisons across time and space. • There is no evidence of a prospective longitudinal study that tracks specific child abuse prevention interventions and their effect on the incidence of EAV • There is growing evidence of an association between ACE and EAV moderated by gender and physical and mental health.

**Response**

**All comments have been addressed in the manuscript.**

**(Page 20):**

Change wording:

- “The current study has provided ~~the base~~ **foundational** evidence **of the need** for more longitudinal studies to examine ~~the trends of~~ the associations between ACEs and EAV, by form or multiple, as well as their moderating factors**,** to understand ~~the specific intervention needed~~ **how** to **better** **serve** the changing needs of older adults and children.
- Policymakers should apply a life course perspective, which propels the need for effective support and services for children and families **who experience victimization and its consequences at all stages of life**~~, as the prevention of adversity in childhood will also positively affect elder abuse prevention~~.”

**Response**

**All comments have been addressed in the manuscript.**
